# The Oral Commensal *Streptococcus mitis* Shows a Mixed Memory Th Cell Signature That Is Similar to and Cross-Reactive with *Streptococcus pneumoniae*

**DOI:** 10.1371/journal.pone.0104306

**Published:** 2014-08-13

**Authors:** Stian André Engen, Håkon Valen Rukke, Simone Becattini, David Jarrossay, Inger Johanne Blix, Fernanda Cristina Petersen, Federica Sallusto, Karl Schenck

**Affiliations:** 1 Department of Oral Biology, University of Oslo, Oslo, Norway; 2 Institute for Research in Biomedicine, Università della Svizzera Italiana, Bellinzona, Switzerland; 3 Department of Periodontology, University of Oslo, Oslo, Norway; Centers for Disease Control & Prevention, United States of America

## Abstract

**Background:**

Carriage of and infection with *Streptococcus pneumoniae* is known to predominantly induce T helper 17 (Th17) responses in humans, but the types of Th cells showing reactivity towards commensal streptococci with low pathogenic potential, such as the oral commensals *S. mitis* and *S. salivarius*, remain uncharacterized.

**Methods:**

Memory CD4^+^ T helper (Th) cell subsets were isolated from healthy human blood donors according to differential expression of chemokine receptors, expanded *in vitro* using polyclonal stimuli and characterized for reactivity against different streptococcal strains.

**Results:**

Th cells responding to *S. mitis, S. salivarius* and *S. pneumoniae* were predominantly in a CCR6^+^CXCR3^+^ subset and produced IFN-γ, and in a CCR6^+^CCR4^+^ subset and produced IL-17 and IL-22. Frequencies of *S. pneumoniae-*reactive Th cells were higher than frequencies of *S. mitis-* and *S. salivarius-*specific Th cells. *S. mitis* and *S. pneumoniae* isogenic capsule knock-out mutants and a *S. mitis* mutant expressing the serotype 4 capsule of *S. pneumoniae* showed no different Th cell responses as compared to wild type strains. *S. mitis*-specific Th17 cells showed cross-reactivity with *S. pneumoniae*.

**Conclusions:**

As Th17 cells partly control clearance of *S. pneumoniae*, cross-reactive Th17 cells that may be induced by commensal bacterial species may influence the immune response, independent of capsule expression.

## Introduction

A reciprocal beneficial relationship has developed between hosts and their symbionts throughout evolution. In the human oral cavity, more than 700 bacterial species can be found [1], [2] of which a healthy person can host more than 200 [3]. In order for the commensals to persist in their niches, it is important that adequate host-microbe interplay is established. This comprises immune exclusion by keeping microbes from interacting with host cell by mucus, SIgA and/or antimicrobial peptides, and immune elimination by innate and adaptive responses without the induction of inflammation [4].

*S. mitis* is a pioneer bacterial species that colonizes the nasopharynx and all sites of the oral cavity from early infancy. Its predominance persists during life and in adults *S. mitis* is found in the oral cavity of nearly all persons. *S. mitis* is closely related to *S. pneumoniae* which also resides in the oronasopharynx: the species may share as much as 39% of their genes, including many of the virulence genes [5]. Despite their genetic similarity, *S. pneumoniae* causes serious infections in about 14.5 million children every year, whereas *S. mitis* rarely causes disease [6].

After the second year of life, a drastic reduction in carriage and disease rate caused by *S. pneumoniae* occurs, independent of capsular serotype [7]. This reduction is attributed to the development of serum IgG and secretory antibodies [8], and to antigen-specific T cell responses [9], [10]. Oral carriage state of *S. mitis* is probably partly regulated by secretion of salivary SIgA [11], [12], but the role of Th cells has not been explored.

Naïve T helper (Th) cells develop into different polarized effector Th subsets that are tailored to effectively cope with the type of infection, including Th1 and Th2 that produce IFN-γ or IL-4, respectively [13]. More recently, Th17 [14], Th22 [15], [16], and Th9 [17] have been described, which produce IL-17, IL-22 or IL-9, respectively. Th cell subsets with a mixed phenotype have been also identified, including T cells producing IL-17 and IFN-γ, or IL-17 and IL-4 [18]–[20]. In this study, we set out to examine the phenotype and functional property of *in vivo*-primed memory CD4^+^ Th cells reactive with antigens from the oral commensal species *S. mitis* and *Streptococcus salivarius*, and compare it to that of *S. pneumoniae*.

Utilizing a T cell screening method, we found that memory Th cells reactive against *S. mitis* and *S. salivarius* are predominantly found in the CCR6^+^ Th1 and Th17 subsets, a distribution similar to that obtained for *S. pneumoniae*. In addition, we observed interspecies cross-reactivity among *S. mitis*–reactive and *S. pneumoniae*-reactive T cell clones.

## Material and methods

### Bacterial strains

Bacterial strains included *S. mitis* (CCUG 31611T, 62644, 62641), *S. pneumoniae* (TIGR4, Sero 1, D39) and *S. salivarius* (JIM8777) (Table 1). Isogenic capsule deletion mutants of *S. mitis* 31611T (*S. mitis* Δ*cps*) and *S. pneumoniae* TIGR4 (*S. pneumoniae* Δ*cps*) and a capsule switch mutant of *S. mitis* 31611T expressing the serotype 4 capsule of *S. pneumoniae* TIGR4 (*S. mitis* 31611T TIGR4) were constructed as described before [21]. All strains were grown in Todd Hewitt Broth (THB) (BD Biosciences, Franklin Lakes, NJ). Over night cultures were diluted in THB and grown to OD = 1 at 600 nm. Cells were harvested by centrifugation at 5000 g for 10 min at 4°C, washed in endotoxin free Dulbecco's-PBS (Sigma-Aldrich, St. Louis, MO) and UV-inactivated for 30 min using UVC 500 Crosslinker (GE Healthcare, Fairfield, CT). The UV-treated bacterial suspensions were aliquoted and frozen at −80°C.

**Table 1:** Streptococcal strains used in this study.

Strains	Description	Source
***S. mitis***		
SK575 (62644)	*Corresponds to CCUG 62644*	CCUG*
SK579 (62641)	*Corresponds to CCUG 62641*	CCUG
CCUG 31611^T^	Wild type S. mitis biovar 1 type strain; corresponds to NCTC12261	CCUG
*S. mitis* Δ*cps*; MI016	CCUG31611^T^ cps::*kan*; Kan^R^	
*S. mitis*_TIGR4_; MI030	CCUG31611^T^ Ω TIGR4 cps locus; Kan^R^, Erm^R^	
***S. pneumoniae***		
D39	Corresponds to NCTC 7466	NCTC**
Serotype 1	Corresponds to sequence type 306	Clinical isolate, Norwegian Institute of Public Health
TIGR4	Wild type serotype 4, transformable strain, sequenced genome	
*S. pneumoniae* Δ*cps*; SP011	TIGR4 cps::*erm*; Erm^R^	
***S. salivarius***		
JIM8777		

*CCUG: Culture collection, University of Göteborg.

**NCTC: National cultures of Type Cultures.

### Blood samples and cell sorting

Blood from anonymized healthy donors was obtained from the Swiss Blood Donation Centers of Basel and Lugano, and used in compliance with the Swiss Federal Office of Public Health (authorization n. A000197/2 to F.S). No submission to a local ethics committee was needed because volunteer donors from the national registry sign an informed consent form (Swiss Red Cross, Medical Questionnaire and Informed Consent Form, version 09), stating that their blood could be used for medical research after definitive anonymization. PBMCs were obtained using Ficoll-Paque PLUS (GE Healthcare) gradient centrifugation. CD14^+^ monocytes and CD4^+^ T cells were isolated by positive selection using magnetic beads (Miltenyi Biotec, Bergisch Gladbach, Germany). CD14^+^ cells were collected and frozen at –80°C for later use. In order to sort distinct Th subsets, CD14^−^CD4^+^ cells were incubated with the following antibodies: anti-CD45RA (Qdot655, Invitrogen, Carlsbad, CA); anti-CXCR3 (APC, BD Biosciences); anti-CCR4 (PE-Cy7, BD Biosciences); anti-CCR6 (Brilliant Violet 605, BioLegend, San Diego, CA); anti-CCR10 (PE, R&D systems, Minneapolis, MN); anti-CD8 (PECy5, Beckman Coulter, Brea, CA); anti-CD19 (PECy5, Beckman Coulter); anti-CD25 (PECy5, Beckman Coulter); anti-CD56 (PECy5, Beckman Coulter). CD45RA^−^CD8^−^CD19^−^CD25^−^CD56^−^ cells were sorted on a FACSAria (BD Biosciences) into the following subsets: i. CXCR3^+^CCR4^−^CCR6^−^CCR10^−^, ii. CCR6^+^CXCR3^+^CCR4^−^CCR10^−^ (both enriched in Th1 and defined thereafter as Th1 and CCR6^+^ Th1, respectively), iii. CCR4^+^CXCR3^−^CCR6^−^CCR10^−^ (enriched in Th2); iv. CCR6^+^CCR4^+^CXCR3^−^CCR10^−^ (Th17), and v. CCR6^+^CCR4^+^CCR10^+^CXCR3^−^ (enriched in Th22). The sorting strategy is summarized in Table S1. Cytokine production by the sorted cell subsets was measured in the 24-hour culture supernatants after activation with immobilized anti-CD3 (clone TR66, 5µg/ml) and anti-CD28 (clone CD28.2; BD Biosciences; 1µgl/ml) antibodies using the cytometric bead array (CBA) (eBiosciences, San Diego, CA), carried out according to the manufacturer's protocol. The characteristics of the subsets, as assayed by cytokine secretion, is shown in Figure S1.

### T cell library construction and screening

T cell libraries were established as described before [22]. Cells were grown in complete media (CM) comprising RPMI-1640 supplemented with 2 mM glutamine, 1% v/v non-essential amino acids, 1% v/v sodium pyruvate, 0.1% v/v 2-mercaptoethanol, penicillin (50 U/mL) and streptomycin (50 µg/mL) (Gibco, Carlsbad, CA), and 5% v/v human serum (Swiss Blood Center), unless stated otherwise. Memory Th cell subsets obtained by cell sorting as described above, were plated in 96-well U-bottom plates (2000 cells/well) and polyclonally stimulated with 1 µg/mL PHA (Sigma-Aldrich), in the presence of irradiated (45 Gy) allogeneic PBMCs and 500 U/mL IL-2. After 7d, the cells were transferred to 24-well plates for further expansion of total 20d. Half of the volume of the medium was changed every other day. For stimulation, the cultures were washed three times in RPMI-1640 with HEPES (Gibco) and 1% v/v FCS, before each well was tested for streptococcal antigen-reactivity by 3d co-culture of 2.5×10^5^ T cells and 2.0×10^4^ irradiated (45 Gy) autologous monocytes, pulsed with whole cell UV-inactivated bacteria (MOI: 100:1) for 5 h. At day 3 of the co-culture, [^3^H]-thymidine was added and proliferation was measured on a beta-counter after 18 h.

### Cross reactivity assay

Wells containing Th17 cells reactive to *S. mitis* and *S. pneumoniae* were cloned by limiting dilution. First, T cells (2.5×10^5^) were stained with CFSE and co-cultured with irradiated (45 Gy) autologous monocytes pulsed with whole cell UV-inactivated bacteria for 5 h. At day 7 of co-culture, CFSE-low proliferating cells were sorted and plated at 0.5 cells/well, stimulated with 1 µg/mL PHA and 0.5×10^4^ irradiated allogeneic PBMCs/well in CM supplemented with 500 U/mL IL-2 in 384-well plates. During 20 d of expansion, proliferating wells were transferred to 96-well U-bottom plates and further to 24-well plates before re-stimulating with whole-cell UV-inactivated bacteria as described above.

### Inhibition assay

Tetanus toxoid (TT)-reactive Th17 memory T cells were sorted and cloned as described above. 2.5×10^4^ T cells were co-cultured with a 2-fold dilution series of irradiated monocytes, ranging from 4×10^4^ to 1.25×10^3^ monocytes/well, and 5 µg/mL TT alone or in the presence of whole cells from *S. mitis* or *S. pneumoniae* (MOI: 100:1). After 3 d in culture, [^3^H]-thymidine was added and proliferation was determined by [^3^H]-thymidine incorporation after 18 h.

### Cytometric bead array (CBA)

To quantify IFN-γ, IL-17A and IL-22, supernatants of CCR6^+^ Th1 and Th17 cells stimulated with *S. mitis* 31611T and *S. pneumoniae* TIGR4 were analyzed using the cytometric bead array (CBA) (eBiosciences, San Diego, CA), carried out according to the manufacturer's protocol.

### Statistical analysis

Student t tests were used to assess differences in cytokine secretion. P values of lower than 0.05 were considered to indicate statistically significant.

## Results

### Circulating T helper memory cells show a heterogeneous signature after stimulation with *S. mitis*, similar to that obtained with *S. pneumoniae*

**Figure 1:**
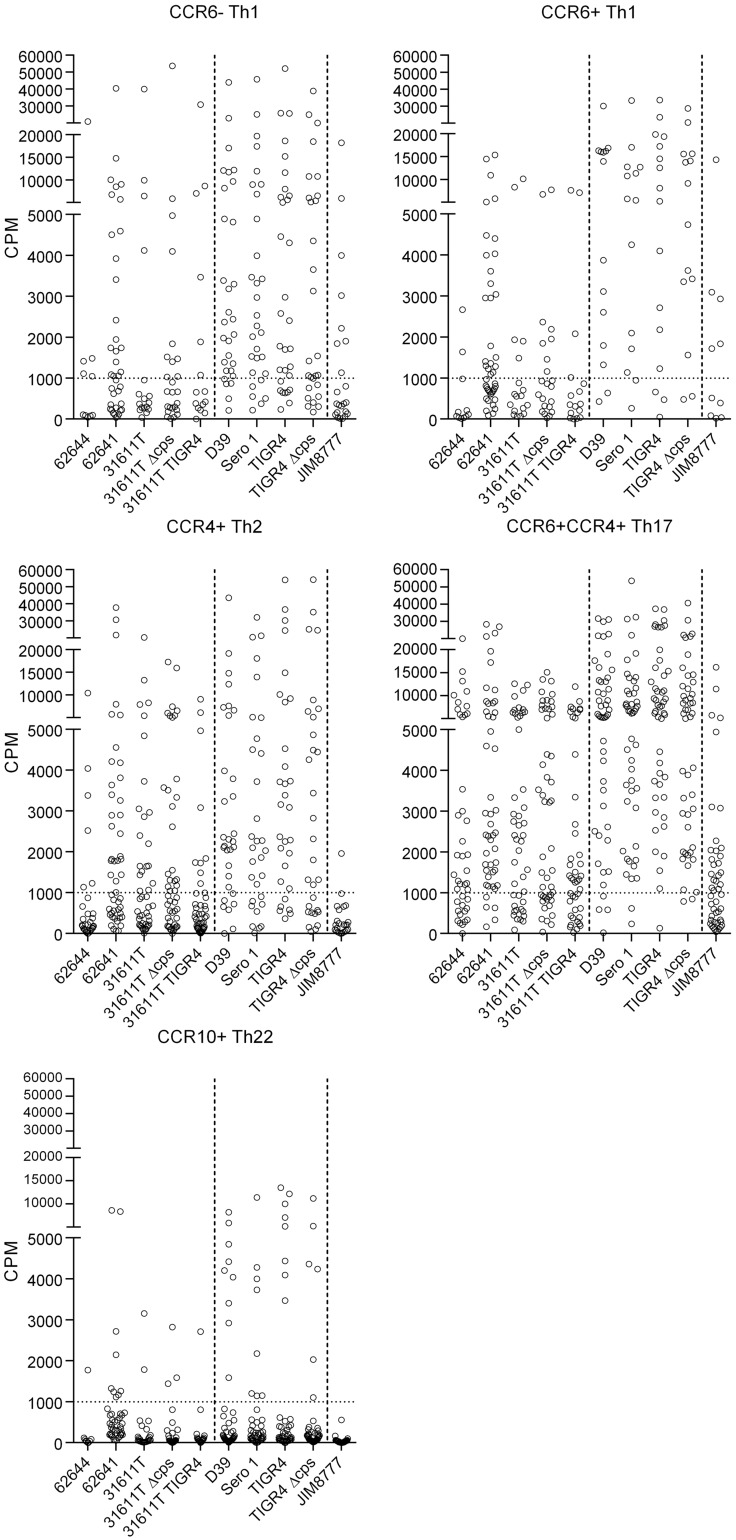
Distribution of antigen-specific memory Th subsets after exposure to streptococcal antigens by autologous monocytes. Raw data of a T cell library of a representative donor screened for reactivity for a panel of ten streptococci. Antigen-specific activity was quantified as a response in increased T cell proliferation determined by thymidine incorporation. Each graph represents the screening of reactivity of one T cell subset to the pane00l of bacteria and each circle represents one well of the respective subset. The dashed line represents lowest counts per minute (CPM) values included in the analysis.

**Figure 2:**
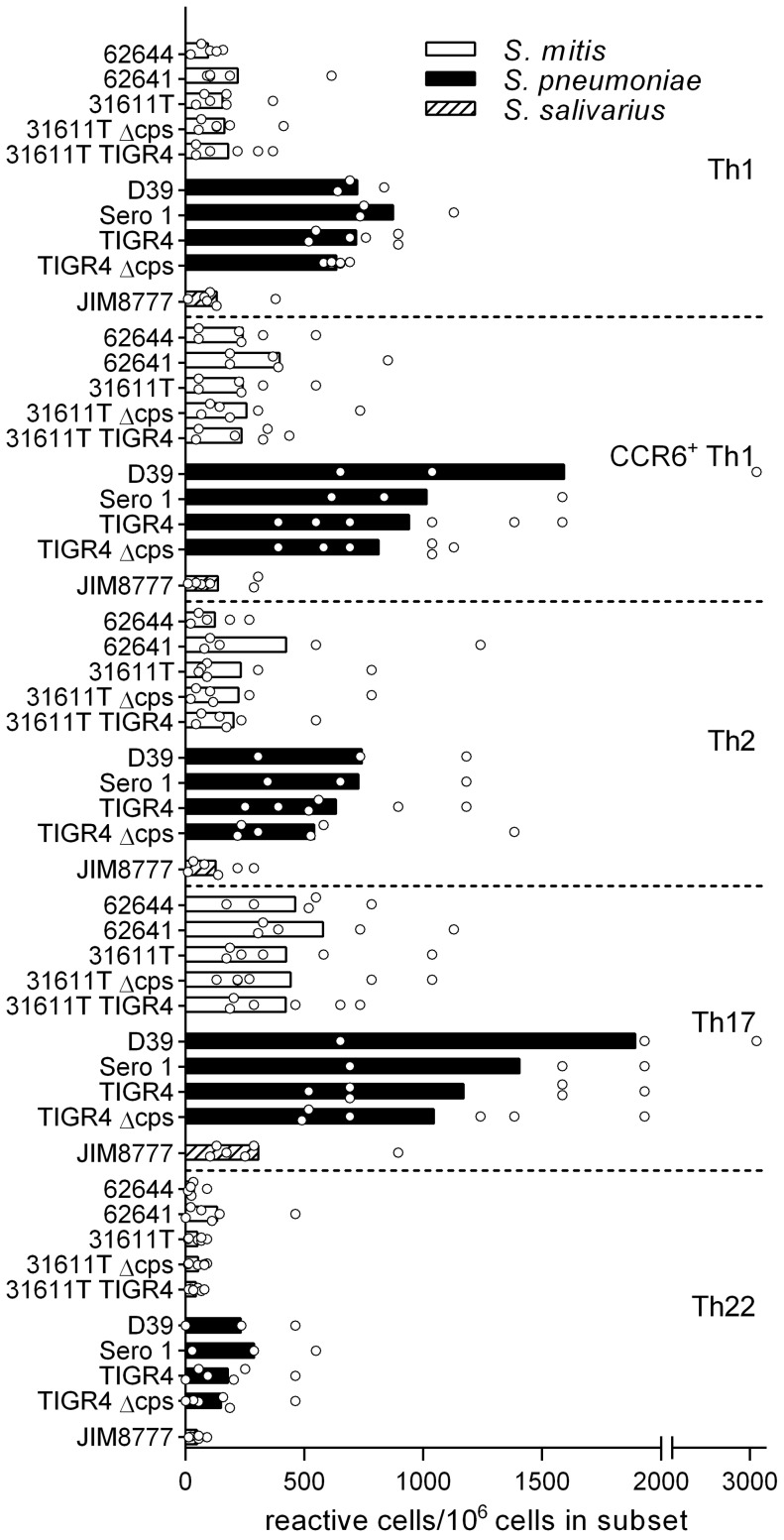
Subset distribution of CD4^+^ memory T cells in response to oropharyngeal-associated streptococcal bacteria. Distribution of single donor (circles) (N  =  3–6) and mean (bars) frequencies of CD4^+^ memory T cells among the CCR6^−^ Th1, CCR6^+^ Th1, Th2, Th17 and Th22 subsets reactive to streptococcal antigens. Data are presented as reactive cells per one million cells in the respective subsets. Open bars: *S. mitis*; closed bars: *S. pneumoniae*; hatched bars: *S. salivarius*. 62644: *S. mitis* CCUG 62644; 62641: *S. mitis* CCUG 62641; 31611T: *S. mitis* CCUG 31611T; 31611T Δcps: *S. mitis* CCUG 31611T capsule deletion mutant; 31611T TIGR4: *S. mitis* CCUG 31611T mutant with capsule from *S. pneumoniae* TIGR4; D39: *S. pneumoniae* D39*;* Sero 1: clinical isolate of *S. pneumoniae* serotype 1; TIGR4: *S. pneumoniae* TIGR4; TIGR4 Δcps: *S. pneumoniae* TIGR4 capsule deletion mutant; JIM8777: *S. salivarius* JIM8777.

Five CD4^+^ memory Th cell subsets from PBMCs of healthy donors were isolated according to the expression of chemokine receptors as described before [22]. For each CD4^+^ T cell subset in each donor, 48 cell lines were established and polyclonally expanded for 16–20 days prior to screening. Each T cell line was then screened for reactivity against autologous monocytes pulsed with three *S. mitis* strains (62644, 62641, 31611T) and responding T cells were detected by [^3^H]-thymidine incorporation. Cultures containing proliferating T cells were identified by incorporation of [^3^H]-thymidine and precursor frequencies were calculated based on the number of negative wells, according to the Poisson distribution and expressed per million cells [23]. A representative example obtained from one of the donors is shown in Figure 1 and the distribution of responding T cells in the different subsets for the 6 donors analyzed is summarized in Figure 2. The raw data and the subset distribution of wells reactive with the streptococcal species for each donor as percentage of the total of all subsets for each strain are shown in Tables S2 and S3, respectively. The frequency of Th17 cells was highest while that of the Th22 cells was lowest for all strains. Intraspecies (*S. mitis* 62644, 62641, 31611T) signatures were similar, but frequencies of memory T cells reactive to *S. mitis* strain 62641 were slightly enhanced in all subsets. The frequencies of Th subsets responsive to the commensal *S. salivarius* were similar to that observed for *S. mitis*.

The signatures were viewed relative to that of *S. pneumoniae,* strains TIGR4, D39, Sero 1, representing serotypes 4, 2, and 1, respectively. The profiles of these three strains were similar in the Th1, Th2 and Th22 subsets but strain D39 displayed a stronger reactivity to the CCR6^+^ Th1 and Th17 subsets (Figure 2). The frequencies of the *S. pneumoniae*-reactive cells were consistently and markedly higher than those for *S. mitis* and *S. salivarius*.

### Capsule expression does not significantly affect the pattern of streptococci-reactive Th cell subset frequencies and relative distribution

*S. pneumoniae* strains are divided into serotypes according to the polysaccharide composition of their capsules. *S. mitis* strains can also express capsule [21]. Deletion of the *S. mitis* capsule or replacing it with capsule from *S. pneumoniae* TIGR4 did not significantly affect reactivities (Figure 1 and 2). In *S. pneumoniae* TIGR4, capsule deletion had no significant effect on Th cell subset frequencies. Capsule from type 1 *S. pneumoniae* strains, such as strain Sero 1, have zwitterionic characteristics and can activate T cell-dependent immune responses [24]. In the present study, however, no significant difference in frequency of Th reactive with type 1 pneumococcus relative to type 2 (D39) or type 4 (TIGR4) was observed (Figure 2).

### *S. mitis* shows a suppressive effect on T cell responses to unrelated antigen

**Figure 3:**
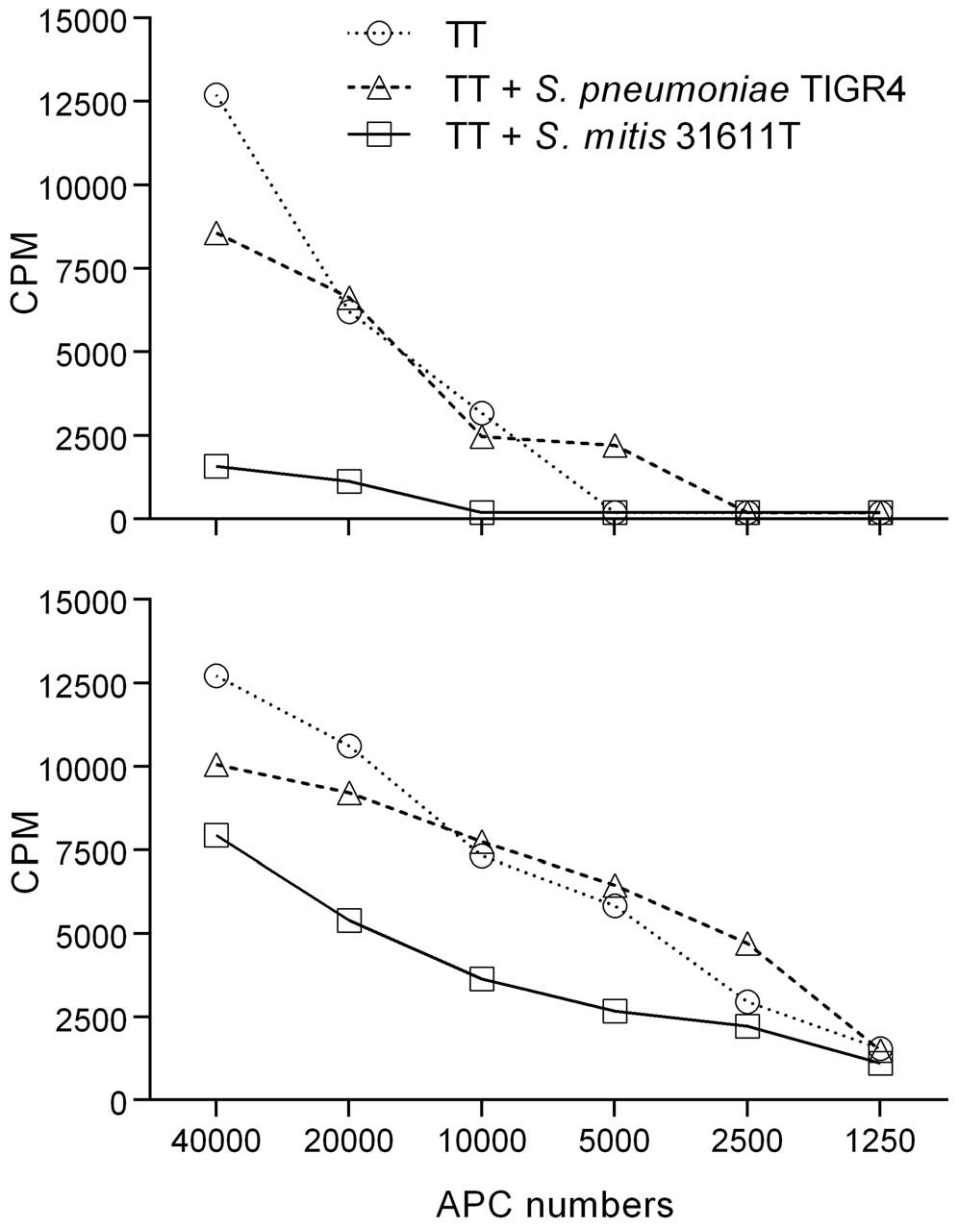
Response of TT-specific Th17 memory cell clones to native antigen in combination with bacterial cells. Tetanus toxoid (TT)-specific CD4^+^ Th17 memory T cell clones were co-cultured with autologous monocytes and either TT alone or TT and *S. mitis* 31611T or *S. pneumoniae* TIGR4 (MOI: 100:1) for 3 d before proliferation was determined by [^3^H]-thymidine incorporation (A: clone 1, B: clone 2).

To investigate if the overall lower Th cell responses observed for *S. mitis* compared to *S. pneumoniae* could be due to antigen-unspecific T cell inhibitory effects of *S. mitis*, tetanus toxoid (TT)-specific Th17 cell clones were co-cultured with autologous monocytes pulsed with TT alone or with TT and *S. mitis* 31611T or *S. pneumoniae* TIGR4. Adding *S. mitis* cells to the co-cultures reduced the ability of the TT-specific T cells to respond to TT, while little effect was observed when *S. pneumoniae* cells were added (Figure 3).

### *S. mitis* and *S. pneumoniae* induce IFN-γ, IL-17 and IL-22 secretion by CCR6^+^ Th1 and Th17 cells

**Figure 4:**
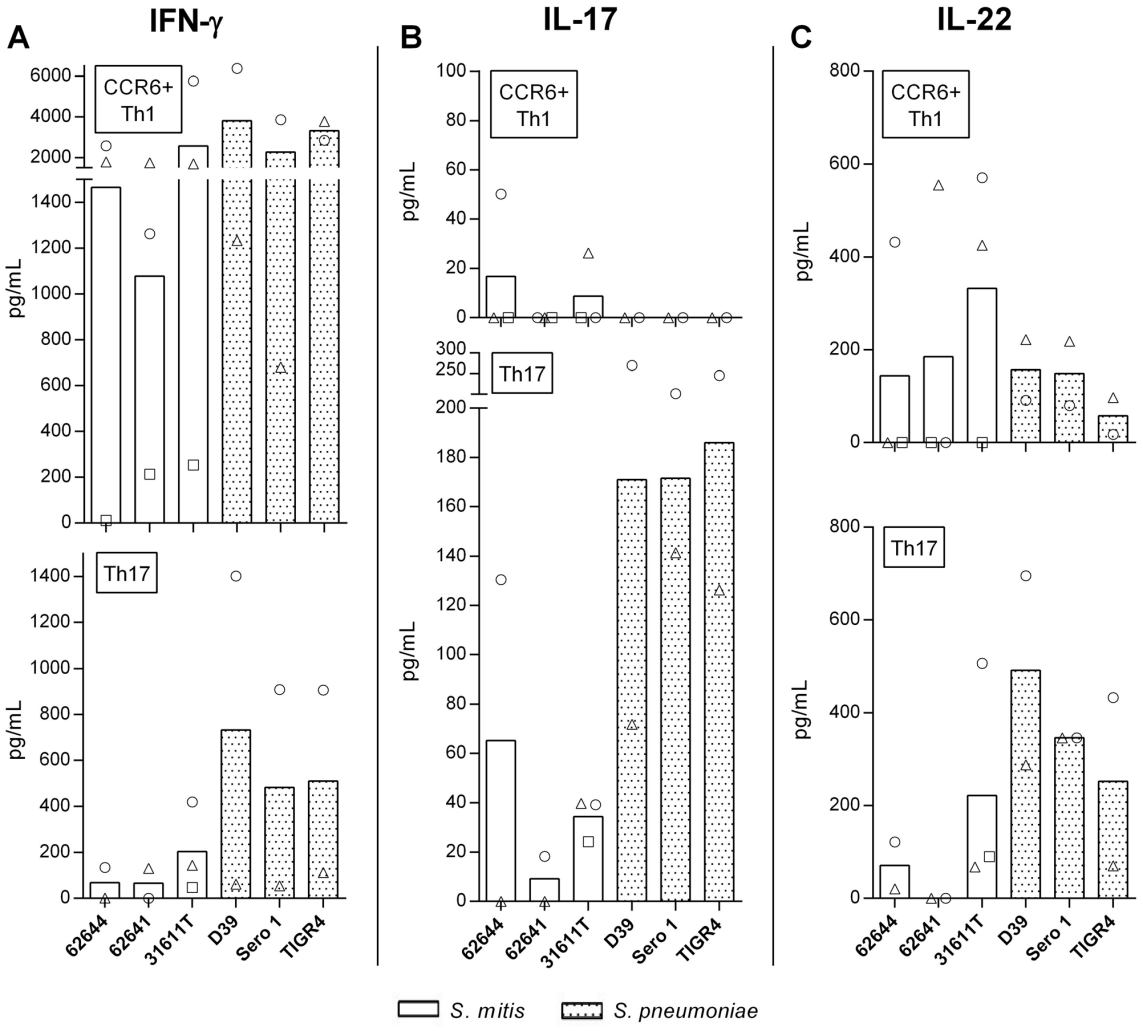
Cytokine production by CCR6^+^ Th1 and Th17 cells in response to streptococci. Quantities of IFN-γ (A), IL-17A (B) and IL-22 (C) were determined in supernatants of CCR6^+^ Th1 and Th17 CD4^+^ memory T cells co-cultured for 3 d with autologous monocytes and whole cell, UV-inactivated *S. mitis* 31611T or *S. pneumoniae* TIGR4 (MOI: 100:1). Bars represent averaged values of different donors (symbols). Th17 cells secreted statistically significantly more IFN-γ, IL-17A and IL-22 when stimulated with *S. pneumoniae* as compared with stimulation with *S. mitis* (Student t test; P < 0.05; see text).

As shown in Figures 1 and 2, the highest response to the streptococcal strains was observed within the CCR6^+^ Th1 and Th17 memory cell subsets. For closer characterization, quantities of signature cytokines, *i.e.* IFN-γ, IL-17A and IL-22, were examined by a cytometric bead array (CBA) in supernatants of the T cell lines co-cultured 3 d with monocytes pulsed with *S. mitis* or *S. pneumoniae* wild types. CCR6^+^ Th1 cells released more IFN-γ and less IL-17 than the Th17 cells (Figure 4). To evaluate the differences in cytokine secretion between cultures stimulated with either *S. mitis* or *S. pneumoniae*, the donors' cytokine secretion data were averaged within each stimulating strain (3 *S. mitis* and 3 *S. pneumoniae* strains; Figure 4). The means for the *S. mitis* and 3 *S. pneumoniae* stimulations were then compared using Student t tests to reveal differences between the species. No statistically significant differences in cytokine secretion were detected within the CCR6^+^ Th1 cells (P>0.05). The Th17 cells stimulated with *S. pneumoniae*, however, released more IFN-γ, IL-17 and IL-22 than those stimulated with *S. mitis* (P<0.05; Figure 4).

### Th17 clones show cross-reactivity for *S. mitis* and *S. pneumoniae*

**Figure 5:**
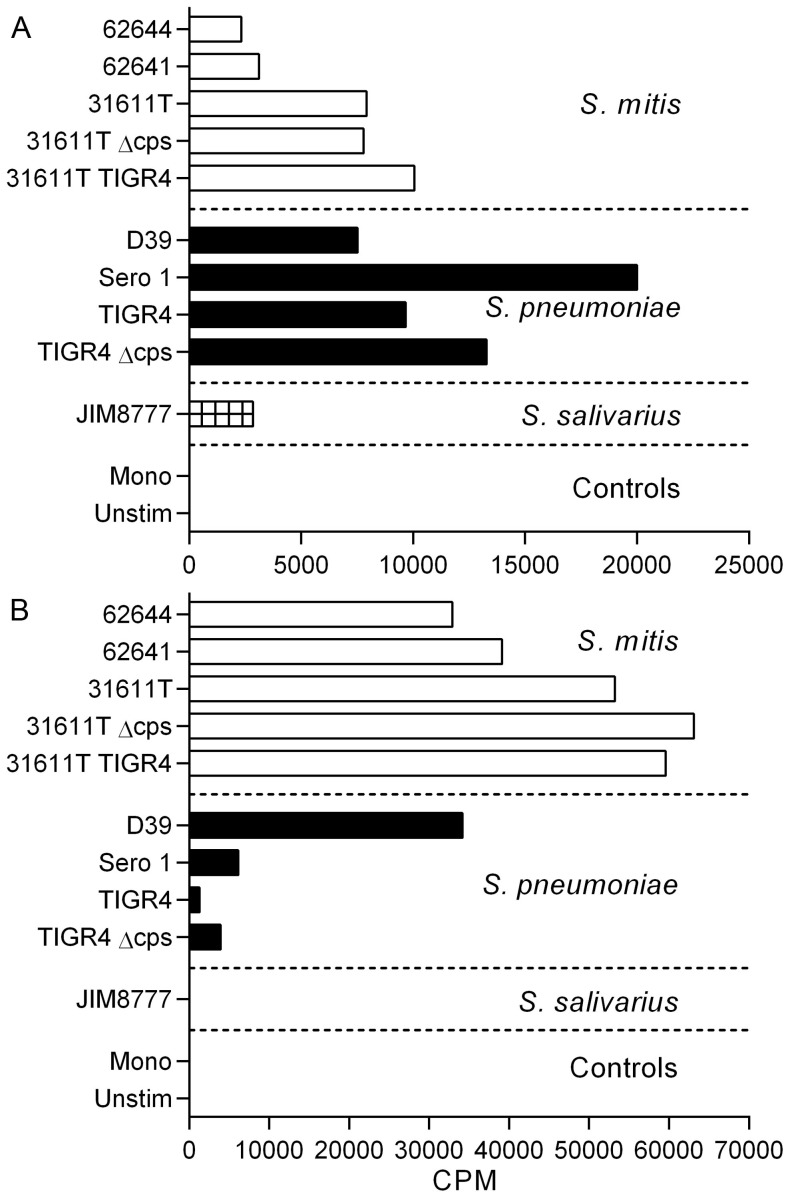
Cross-reactivity of CD4^+^ Th17 memory T cell clones in response to oropharyngeal-associated streptococcal strains. Th17 cells from wells initially reactive to *S. mitis* 62641 (A) and *S. pneumoniae* D39 (B) were cloned by limiting dilution and distribution of intra- and interspecies cross-reactivity was determined by thymidine incorporation. Bars represent single re-stimulation of each clone and data are presented as counts per minute (CPM).

As similar T cell subset patterns were found for *S. mitis* and *S. pneumoniae* and as the library comprised wells that showed reactivity to both *S. mitis* and *S. pneumoniae* strains, we examined whether this could be due to cross-reactive T cells. Cells from Th17 wells that were reactive with *S. mitis* 62641 or *S. pneumoniae* D39 in the initial stimulation were cloned and re-stimulated with the panel of bacterial strains. Clones initially reactive to *S. mitis* showed both inter- and intraspecies reactivity (Figure 5A), indicating cross-reactivity, while clones initially reactive to *S. pneumoniae* showed considerable cross-reactivity to all *S. mitis* strains (Figure 5B).

## Discussion

Little is known about the subsets of Th cells responsive to commensal bacteria [25]. Here, we used a high-throughput T cell library method to map the Th cell signature that recognizes antigens from *S. mitis* and *S. salivarius*. CD4^+^CD45RA^−^ memory Th cells were sorted into subsets based on differential chemokine receptor expression. This sorting was based on the knowledge of a co-regulation of effector function and migratory properties during T cell differentiation, a mechanism which ensures selective recruitment of different effector T cells to inflamed tissues in response to inflammatory chemoattractants [26]. Compared with *in vitro* antigen stimulation of unfractionated T cells and subsequent phenotyping, sorting of the cells before stimulation has the advantage of establishing the *in vivo* subsets' identity before the cells are brought into culture, ensuring an “untouched” phenotype. We chose to expose the T cell cultures to whole, UV-inactivated bacterial cells to avoid compromising the integrity of surface molecules by unfavorable thermal or chemical conditions.

The primary aim of this study was to determine the distribution of Th subsets that recognize antigens from the ubiquitous oral commensal *S. mitis* in healthy donors. We found that the numbers of *S. mitis*- and *S. salivarius*-reactive Th cells were heterogeneously distributed among the five subsets tested, but with a predominance of CCR6^+^ Th1 and Th17 cells. *S. pneumoniae* is genetically closely related to *S. mitis* and was therefore included in the study. As *S. pneumoniae* has a marked pathogenic potential and *S. mitis* seldom causes disease, different distributions of response in the Th subset signature might be expected, but the results obtained showed that the Th response reactive with *S. pneumoniae* is strikingly similar to that of *S. mitis*. The signature and frequencies of responding cells to *S. salivarius* also coincided with that of *S. mitis*. The prominent Th17 response to *S. pneumoniae* is in accordance with studies on human lymphoid tonsillar tissue [27] and bronchoalveolar lavage [28], which show Th1/Th17- and IL-17A-dependent pneumococcal clearance, respectively.

The CCR6^+^ Th1 subset is a recently described Th subset that has characteristics of both Th1 and Th17 subsets. Th1 and Th17 lymphocytes are characterized by specific transcription factors, surface receptors and cytokine secretion (Th1: T-bet, CXCR3, and IFN-γ; Th17: RORC, CCR4/CCR6, and IL-17). Upon polyclonal stimulation, the intermediate CCR6^+^ Th1 subset can secrete both IL-17 and IFN-γ and express RORC and T-bet ([8], [18], [29], [30]. Expression of the Th17 surface marker CD161 and shared TCR clonality indicate that CCR6^+^ Th1 cells are of Th17 ancestry [29]. Furthermore, Th17 cells exposed to Th1-polarizing conditions convert to the intermediate CCR6^+^ Th1 cells (RORC^+^, T-bet^+^, IL-17^+^ IFN-γ^+^), while Th1 cells exposed to Th17-polarizing conditions remain Th1 cells [29], [30]. A proportion of Th1 cells are thought to be of Th17 origin due to expression of factors characteristic for Th17 (RORC, CCR6, CD161) [29]. In contrast to polyclonal stimulation, however, exposure of CCR6^+^ Th1 cells to *Mycobacterium tuberculosis* PPD revealed an antigen-specific response characterized by absence of IL-17 secretion [18]. Our present observation that the CCR6^+^ Th1 cell response to the streptococcal strains tested produced IFN-γ but not IL-17 complies with this observation [18].

The CCR6^+^ Th1 and Th17 subsets presently examined produced a mixture of IFN-γ, IL-17 and IL-22 in response to streptococcal stimulation, effector cytokines that can be involved in the clearance of *S. pneumoniae* in humans. IFN-γ and IL-17 support macrophage and neutrophil defenses, respectively [26]. In a human infection model, carriage of *S. pneumoniae* increased the prevalence of CD4^+^IL-17A^+^TNF^+^IFN-γ^+^ Th cells in lungs and blood, as compared to non-carriers [28]. Another study showed an IFN-γ-dependent but IL-17-independent clearance of invading *S. pneumoniae* [31]. Finally, significant increases in proportions of IFN-γ^+^ CD4^+^ cells were seen in patients with community-acquired pneumonia [32]. IL-22 promotes epithelial proliferation, expression of antimicrobial proteins involved in host defense in the skin, airways and intestine, and production of inflammatory mediators and chemokines from epithelial cells [33]. Knock-out of IL-22 renders mice more susceptible to infection with *S. pneumoniae* than wild type animals [34]. IL-22 by itself has protective and regenerative functions, but together with IL-17, it supports inflammation [33]. Here, we observed that Th17 cells secreted significantly higher amounts of IFN-γ, IL-17 and IL-22 when challenged with *S. pneumoniae* species as compared with *S. mitis*. This suggests that *S. pneumoniae* can induce a more pronounced inflammatory response as compared with *S. mitis*.

A consistently lower frequency of Th cells responsive to *S. mitis* compared to *S. pneumoniae* was observed. The finding that *S. mitis* reduced proliferation of T cell clones specific for an unrelated antigen (TT) suggests an inhibitory effect of *S. mitis.* This inhibition can either be on the APCs directly by preventing activation and/or antigen presentation, or directly on the memory Th cells by interfering with the APC-T cell interaction. TT-induced T cell proliferation was sustained upon addition of *S. pneumoniae* which indicated that the inhibitory effect is not a shared trait between *S. mitis* and *S. pneumoniae*, but specific for *S. mitis*. Another cause for the higher frequencies of *S. pneumoniae*-specific cells as compared with those for *S. mitis* can be the occurrence of more species-specific immunogenic antigens in the former species.

Expression of capsule is a hallmark of virulence of *S. pneumoniae* and the capsules comprise different serotypes that are important for the species to evade immune responses [35]. Capsule antigens comprise multi-epitopic repetitive carbohydrate units and have been considered as T cell-independent antigens, with exception of that from *S. pneumoniae* type 1 (Sp1) that has zwitterionic properties, and is capable of activating T cells in an MHC class II-dependent manner [24], [36]. Recently, *S. mitis* also has been shown to express capsule [21]. We tested isogenic capsule deletion mutants of *S. mitis* and *S. pneumoniae* but little difference in Th cell signatures was observed as compared with wild type strains. Replacing native capsule of *S. mitis* 31611T with capsule of *S. pneumoniae* TIGR4 neither altered the Th cell responses relative to the *S. mitis* wild type. This indicates that other factors than capsule expression are responsible for the raised frequencies of antigen-specific Th cells to *S. pneumoniae* as compared with *S. mitis*. The present human study support previous findings showing that murine splenocytes from animals injected with whole-cell vaccine induce high IL-17 responses, independent of presence or type of capsule [37]. In addition, the lack of differences in Th cell responses to T cell-dependent capsule serotype (Sp1) and T cell-independent capsule serotype (*S. pneumoniae* TIGR4) supports the notion that capsule is not recognized by CD4^+^ memory T cells *in vivo.*

Heterologous immunity, the immunity that can develop to one pathogen after a host has been exposed to non-identical pathogens, has been closely studied in viral diseases [38], but less is known in bacterial infections. It can be mediated by specific cross-reactive T cells or antibodies, but can also be less specific [38]. We observed many cultures containing Th17 cells reactive for *S. mitis* and *S. pneumoniae* and hypothesized this could be due to cross-reactive cells. Indeed, Th cell clones from cultures responsive to *S. mitis* showed both intra- and inter-species cross-reactivity. Recently, 12 immunogenic Th17 antigens were isolated from soluble fractions of *S. pneumoniae* cell extracts [37]. We inspected the genomes of the three *S. mitis* strains used in this study and this revealed homologues to the 12 prominent T cell antigens from *S. pneumoniae* (90 to 100% identity) in strains 62644 and 62641 (data not shown). In the *S. mitis* type strain 31611T, 11 of the antigens were found. This means that the current cross-reactivity findings can be due to antigens common to *S. mitis* and *S. pneumoniae*. In the present investigation, it is not possible to identify the antigenic origin of any clones, but the existence of Th cell clones cross-reactive for the commensal *S. mitis* and the pathogenic *S. pneumoniae* can mean that immunologic memory induced by exposure to *S. mitis* or other related commensal streptococci can affect both carriage and clearance of *S. pneumoniae* since it is known that *S. pneumoniae*–specific Th17 cells play a role in these processes [28].

In conclusion, the similar and cross-reactive T memory cell responses against *S. mitis* and *S. pneumoniae* indicate that the species have the potential to influence their mutual colonization. It is possible that carriage distributions of *S. mitis* strains will be shown to affect the performance of future experimental pneumococcal vaccine formulations.

## Supporting Information

Figure S1 **Cytokine secretion within Th subsets.** Th subsets were sorted as described in Material and Methods and cytokine secretion in supernatant was measured by the CBA method after polyclonal stimulation with anti-CD3/anti-CD28 for 24 h. Bars represent averaged values of three samples and flags indicate standard deviation.
(DOCX)

Table S1 Overview of surface markers used for sorting of CD45RA^-^CD4^+^ Th subsets.
(DOCX)

Table S2 Numbers of T cells reactive with streptococci per 1×10^6^ T cells per subset per donor.
(DOCX)

Table S3 Wells reactive with streptococci as percentage of total number of wells for all subsets within each strain.
(DOCX)
