# Supplementary material for: The Oral Commensal Streptococcus mitis Shows a Mixed Memory Th Cell Signature That Is Similar to and Cross-Reactive with Streptococcus pneumoniae
Source: PLoS One. 2014 Aug 13;9(8):e104306. doi: 10.1371/journal.pone.0104306 (PMC4131883; doi:10.1371/journal.pone.0104306)

**Figure S1. Cytokine secretion within Th subsets.** Th subsets were sorted as described in Material and Methods and cytokine secretion in supernatant was measured by the CBA method after polyclonal stimulation with anti-CD3/anti-CD28 for 24 h. Bars represent averaged values of three samples and flags indicate standard deviation.


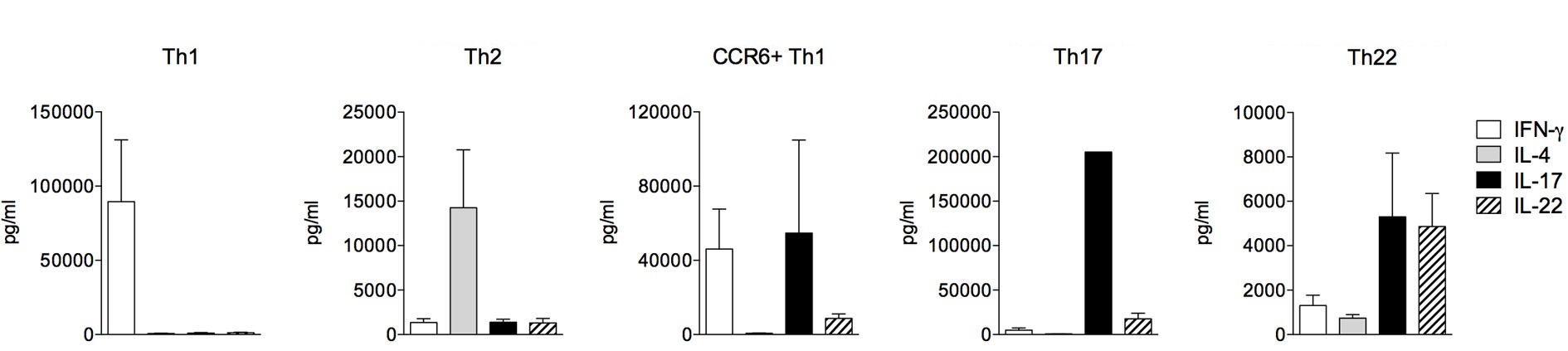

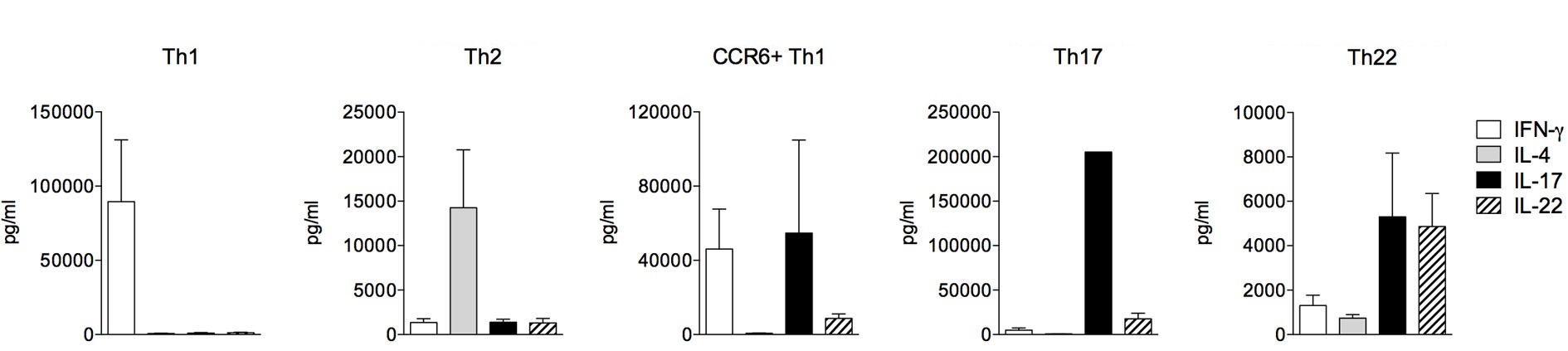

Supplement: Figure S1 — Cytokine secretion within Th subsets. Th subsets were sorted as described in Material and Methods and cytokine secretion in supernatant was measured by the CBA method after polyclonal stimulation with anti-CD3/anti-CD28 for 24 h. Bars represent averaged values of three samples and flags indicate standard deviation. (DOCX) [file pone.0104306.s001.docx]
