# Supplementary material for: The Oral Commensal Streptococcus mitis Shows a Mixed Memory Th Cell Signature That Is Similar to and Cross-Reactive with Streptococcus pneumoniae
Source: PLoS One. 2014 Aug 13;9(8):e104306. doi: 10.1371/journal.pone.0104306 (PMC4131883; doi:10.1371/journal.pone.0104306)
Supplement: Table S1 — Overview of surface markers used for sorting of CD45RA-CD4+ Th subsets. (DOCX) [file pone.0104306.s002.docx]

**Table S1. Overview of surface markers used for sorting of CD45RA^-^CD4^+^ Th subsets.**

|  | CD45RA | CD8 | CD19 | CD25 | CD56 | CXCR3 | CCR4 | CCR6 | CCR10 |
| --- | --- | --- | --- | --- | --- | --- | --- | --- | --- |
| Th1 | - | - | - | - | - | **+** | - | - | - |
| CCR6^+^ Th1 | - | - | - | - | - | **+** | - | **+** | - |
| Th2 | - | - | - | - | - | - | **+** | - | - |
| Th17 | - | - | - | - | - | - | **+** | **+** | - |
| Th22 | - | - | - | - | - | - | + | + | + |
