# Supplementary material for: The Oral Commensal Streptococcus mitis Shows a Mixed Memory Th Cell Signature That Is Similar to and Cross-Reactive with Streptococcus pneumoniae
Source: PLoS One. 2014 Aug 13;9(8):e104306. doi: 10.1371/journal.pone.0104306 (PMC4131883; doi:10.1371/journal.pone.0104306)
Supplement: Table S2 — Numbers of T cells reactive with streptococci per 1×10^6^ T cells per subset per donor. (DOCX) [file pone.0104306.s003.docx]

**Table S2. Numbers of T cells reactive with streptococci per 1 x 10^6^ T cells per subset per donor.**

|  | Donor | Th1 | CCR6^+^ Th1 | Th2 | Th17 | Th22 |
| --- | --- | --- | --- | --- | --- | --- |
| *S. mitis*  62644 | 1 | 67 | 226 | 55 | 172 | 26 |
|  | 2 | 104 | 235 | 91 | 288 | 11 |
|  | 3 | 158 | 326 | 21 | 549 | 91 |
|  | 4 | 130 | 549 | 187 | 519 | 32 |
|  | 5 | 67 | 32 | 0 | 11 | 14 |
|  | 6 | 67 | 44 | 79 | 463 | 11 |
| *S. mitis*  62641 | 1 | 616 | 852 | 549 | 737 | 112 |
|  | 2 | 104 | 187 | 79 | 307 | 21 |
|  | 3 | 91 | 187 | 104 | 390 | 144 |
|  | 4 | 104 | 390 | 144 | 326 | 67 |
|  | 5 | 11 | 11 | 11 | 11 | ND |
|  | 6 | 187 | 368 | 1242 | 1131 | 463 |
| *S. mitis*  31611T | 1 | 104 | 226 | 91 | 187 | 53 |
|  | 2 | 172 | 235 | 91 | 235 | 11 |
|  | 3 | 172 | 326 | 67 | 326 | 67 |
|  | 4 | 368 | 549 | 307 | 582 | 91 |
|  | 5 | 79 | 55 | 55 | 172 | 14 |
|  | 6 | 44 | 55 | 784 | 1040 | 67 |
| *S. mitis*  31611T Δcps | 1 | 130 | 144 | 117 | 219 | 53 |
|  | 2 | 130 | 187 | 104 | 219 | 11 |
|  | 3 | 187 | 307 | 44 | 269 | 91 |
|  | 4 | 413 | 737 | 269 | 784 | 79 |
|  | 5 | 67 | 67 | 21 | 130 | 14 |
|  | 6 | 55 | 104 | 784 | 1040 | 79 |
| *S. mitis*  31611T TIGR4 | 1 | 104 | 208 | 144 | 187 | 53 |
|  | 2 | 219 | 326 | 172 | 288 | 21 |
|  | 3 | 368 | 438 | 67 | 463 | 67 |
|  | 4 | 307 | 347 | 235 | 653 | 79 |
|  | 5 | 44 | 55 | 44 | 203 | 14 |
|  | 6 | 44 | 44 | 549 | 737 | 32 |
| *S. pneumoniae* D39 | 1 | ND | ND | ND | ND | ND |
|  | 2 | ND | ND | ND | ND | ND |
|  | 3 | ND | ND | ND | ND | ND |
|  | 4 | 837 | 3087 | 737 | 3087 | 235 |
|  | 5 | 693 | 653 | 307 | 653 | ND |
|  | 6 | 640 | 1040 | 1184 | 1936 | 463 |
| *S. pneumoniae* Serotype 1 | 1 | ND | ND | ND | ND | ND |
|  | 2 | ND | ND | ND | ND | ND |
|  | 3 | ND | ND | ND | ND | ND |
|  | 4 | 1131 | 1589 | 653 | 1589 | 288 |
|  | 5 | 737 | 616 | 347 | 693 | 29 |
|  | 6 | 752 | 837 | 1184 | 1936 | 549 |
| *S. pneumoniae* TIGR4 | 1 | 896 | 693 | 390 | 693 | 95 |
|  | 2 | 693 | 1589 | 560 | 519 | 55 |
|  | 3 | 519 | 549 | 519 | 1589 | 203 |
|  | 4 | 896 | 1386 | 896 | 1589 | 252 |
|  | 5 | 549 | 390 | 252 | 693 | ND |
|  | 6 | 760 | 1040 | 1184 | 1936 | 463 |
| *S. pneumoniae* TIGR4 Δcps | 1 | 582 | 693 | 235 | 693 | 55 |
|  | 2 | 616 | 1040 | 528 | 519 | 32 |
|  | 3 | 653 | 582 | 219 | 1386 | 187 |
|  | 4 | 616 | 1131 | 582 | 1242 | 158 |
|  | 5 | 653 | 390 | 307 | 490 | ND |
|  | 6 | 693 | 1040 | 1386 | 1936 | 463 |
| *S. salivarius* JIM8777 | 1 | 104 | 67 | 79 | 130 | 55 |
|  | 2 | 130 | 307 | 137 | 288 | 21 |
|  | 3 | 79 | 104 | 11 | 172 | 91 |
|  | 4 | 91 | 44 | 219 | 252 | 55 |
|  | 5 | 11 | 11 | 32 | 104 | 14 |
|  | 6 | 379 | 288 | 288 | 896 | 55 |

ND = not determined
