# Supplementary material for: The Oral Commensal Streptococcus mitis Shows a Mixed Memory Th Cell Signature That Is Similar to and Cross-Reactive with Streptococcus pneumoniae
Source: PLoS One. 2014 Aug 13;9(8):e104306. doi: 10.1371/journal.pone.0104306 (PMC4131883; doi:10.1371/journal.pone.0104306)
Supplement: Table S3 — Wells reactive with streptococci as percentage of total number of wells for all subsets within each strain. (DOCX) [file pone.0104306.s004.docx]

**Table S3. Wells reactive with streptococci as percentage of total number of wells for all subsets within each strain.**

|  | Donor | Th1 | CCR6^+^ Th1 | Th2 | Th17 | Th22 |
| --- | --- | --- | --- | --- | --- | --- |
| *S. mitis*  62644 | 1 | 19.4 | 16.1 | 16.1 | 45.2 | 3.2 |
|  | 2 | 17.6 | 23.5 | 15.7 | 41.2 | 2.0 |
|  | 3 | 18.1 | 23.6 | 2.8 | 44.4 | 11.1 |
|  | 4 | 13.9 | 24.1 | 19.0 | 39.2 | 3.8 |
|  | 5 | 66.7 | 16.7 | 0.0 | 8.3 | 8.3 |
|  | 6 | 3.1 | 3.1 | 31.3 | 59.4 | 3.1 |
| *S. mitis*  62641 | 1 | 23.8 | 25.2 | 22.4 | 25.9 | 2.8 |
|  | 2 | 16.4 | 27.3 | 12.7 | 40.0 | 3.6 |
|  | 3 | 11.4 | 21.4 | 12.9 | 37.1 | 17.1 |
|  | 4 | 11.8 | 34.2 | 15.8 | 30.3 | 7.9 |
|  | 5 | 25.0 | 25.0 | 25.0 | 25.0 | 0.0 |
|  | 6 | 9.6 | 16.0 | 28.2 | 27.6 | 18.6 |
| *S. mitis*  31611T | 1 | 18.0 | 32.0 | 16.0 | 30.0 | 4.0 |
|  | 2 | 23.7 | 30.5 | 13.6 | 30.5 | 1.7 |
|  | 3 | 19.4 | 31.9 | 8.3 | 31.9 | 8.3 |
|  | 4 | 20.8 | 26.7 | 18.3 | 27.5 | 6.7 |
|  | 5 | 21.9 | 15.6 | 15.6 | 43.8 | 3.1 |
|  | 6 | 4.2 | 5.3 | 40.0 | 44.2 | 6.3 |
| *S. mitis*  31611T Δcps | 1 | 21.6 | 21.6 | 19.6 | 33.3 | 3.9 |
|  | 2 | 20.8 | 28.3 | 17.0 | 32.1 | 1.9 |
|  | 3 | 21.7 | 31.9 | 5.8 | 29.0 | 11.6 |
|  | 4 | 20.9 | 28.7 | 15.5 | 29.5 | 5.4 |
|  | 5 | 23.1 | 23.1 | 7.7 | 42.3 | 3.8 |
|  | 6 | 5.0 | 8.9 | 37.6 | 41.6 | 6.9 |
| *S. mitis*  31611T TIGR4 | 1 | 17.0 | 28.3 | 22.6 | 28.3 | 3.8 |
|  | 2 | 22.1 | 29.9 | 18.2 | 27.3 | 2.6 |
|  | 3 | 26.6 | 29.8 | 6.4 | 30.9 | 6.4 |
|  | 4 | 20.8 | 22.6 | 17.0 | 33.0 | 6.6 |
|  | 5 | 13.3 | 16.7 | 13.3 | 53.3 | 3.3 |
|  | 6 | 5.0 | 5.0 | 40.0 | 46.3 | 3.8 |
| *S. pneumoniae* D39 | 1 | ND | ND | ND | ND | ND |
|  | 2 | ND | ND | ND | ND | ND |
|  | 3 | ND | ND | ND | ND | ND |
|  | 4 | 20.5 | 25.2 | 19.5 | 25.2 | 9.5 |
|  | 5 | 28.1 | 27.3 | 17.2 | 27.3 | 0.0 |
|  | 6 | 17.9 | 9.7 | 20.0 | 32.4 | 20.0 |
| *S. pneumoniae* Serotype 1 | 1 | ND | ND | ND | ND | ND |
|  | 2 | ND | ND | ND | ND | ND |
|  | 3 | ND | ND | ND | ND | ND |
|  | 4 | 22.5 | 24.1 | 18.3 | 24.1 | 11.0 |
|  | 5 | 27.8 | 25.6 | 18.0 | 27.1 | 1.5 |
|  | 6 | 18.8 | 8.7 | 19.5 | 31.5 | 21.5 |
| *S. pneumoniae* TIGR4 | 1 | 33.6 | 10.1 | 21.8 | 30.3 | 4.2 |
|  | 2 | 24.2 | 30.9 | 20.8 | 20.8 | 3.4 |
|  | 3 | 19.9 | 20.5 | 19.9 | 29.5 | 10.3 |
|  | 4 | 21.1 | 23.7 | 21.1 | 24.2 | 10.0 |
|  | 5 | 28.3 | 23.0 | 16.8 | 31.9 | 0.0 |
|  | 6 | 17.4 | 9.7 | 20.1 | 32.6 | 20.1 |
| *S. pneumoniae* TIGR4 Δcps | 1 | 32.4 | 11.8 | 17.6 | 35.3 | 2.9 |
|  | 2 | 24.3 | 30.0 | 21.4 | 22.1 | 2.1 |
|  | 3 | 24.1 | 22.8 | 11.7 | 31.0 | 10.3 |
|  | 4 | 20.4 | 25.7 | 19.8 | 26.3 | 7.8 |
|  | 5 | 31.0 | 23.0 | 19.5 | 26.5 | 0.0 |
|  | 6 | 16.7 | 9.7 | 20.8 | 32.6 | 20.1 |
| *S. salivarius* JIM8777 | 1 | 28.1 | 6.3 | 21.9 | 34.4 | 9.4 |
|  | 2 | 16.4 | 32.8 | 16.4 | 31.3 | 3.0 |
|  | 3 | 17.9 | 23.1 | 2.6 | 35.9 | 20.5 |
|  | 4 | 15.1 | 7.5 | 32.1 | 35.8 | 9.4 |
|  | 5 | 6.7 | 6.7 | 20.0 | 60.0 | 6.7 |
|  | 6 | 20.5 | 8.4 | 16.9 | 48.2 | 6.0 |

ND = not determined
